# Comprehensive analysis and identification of subtypes and hub genes of high immune response in lung adenocarcinoma

**DOI:** 10.1186/s12890-024-03130-6

**Published:** 2024-07-04

**Authors:** Han Li, Yuting Lei, Xianwen Lai, Ruina Huang, Yuanyuan Xiang, Zhao Zhao, Zhenfu Fang, Tianwen Lai

**Affiliations:** Department of Respiratory and Critical Care Medicine, The First Dongguan Affiliated Hospital, Guangdong Medical University, Dongguan, 523121 China

**Keywords:** Lung adenocarcinoma, CD8 + T cells, Immune subtype, PD-L1, Immunotherapy response

## Abstract

**Background:**

The advent of immunotherapy targeting immune checkpoints has conferred significant clinical advantages to patients with lung adenocarcinoma (LUAD); However, only a limited subset of patients exhibit responsiveness to this treatment. Consequently, there is an imperative need to stratify LUAD patients based on their response to immunotherapy and enhance the therapeutic efficacy of these treatments.

**Methods:**

The differentially co-expressed genes associated with CD8 + T cells were identified through weighted gene co-expression network analysis (WGCNA) and the Search Tool for the Retrieval of Interacting Genes (STRING) database. These gene signatures facilitated consensus clustering for TCGA-LUAD and GEO cohorts, categorizing them into distinct immune subtypes (C1, C2, C3, and C4). The Tumor Immune Dysfunction and Exclusion (TIDE) model and Immunophenoscore (IPS) analysis were employed to assess the immunotherapy response of these subtypes. Additionally, the impact of inhibitors targeting five hub genes on the interaction between CD8 + T cells and LUAD cells was evaluated using CCK8 and EDU assays. To ascertain the effects of these inhibitors on immune checkpoint genes and the cytotoxicity mediated by CD8 + T cells, flow cytometry, qPCR, and ELISA methods were utilized.

**Results:**

Among the identified immune subtypes, subtypes C1 and C3 were characterized by an abundance of immune components and enhanced immunogenicity. Notably, both C1 and C3 exhibited higher T cell dysfunction scores and elevated expression of immune checkpoint genes. Multi-cohort analysis of Lung Adenocarcinoma (LUAD) suggested that these subtypes might elicit superior responses to immunotherapy and chemotherapy. In vitro experiments involved co-culturing LUAD cells with CD8 + T cells and implementing the inhibition of five pivotal genes to assess their function. The inhibition of these genes mitigated the immunosuppression on CD8 + T cells, reduced the levels of PD1 and PD-L1, and promoted the secretion of IFN-γ and IL-2.

**Conclusions:**

Collectively, this study delineated LUAD into four distinct subtypes and identified five hub genes correlated with CD8 + T cell activity. It lays the groundwork for refining personalized therapy and immunotherapy strategies for patients with LUAD.

**Supplementary Information:**

The online version contains supplementary material available at 10.1186/s12890-024-03130-6.

## Introduction

Lung cancer, the most prevalent form of cancer, presents a significant threat to human health. It is broadly classified into two subtypes: Non-Small Cell Lung Cancer (NSCLC) and Small Cell Lung Cancer (SCLC). NSCLC, encompassing lung adenocarcinoma (LUAD) and squamous cell carcinoma (LUSC), represents approximately 80% of all lung cancer cases [1–3]. Despite advancements in various therapeutic methods, including surgery, LUAD’s five-year survival rate remains discouragingly low [4]. The advent of immunotherapy, particularly through the use of immune checkpoint inhibitors (ICIs) targeting PD-1 and PD-L1, has heralded a new era in lung cancer treatment [5]. Nowadays, immunotherapy is employed across a spectrum of cancer types, lung cancer included [6–8]. However, the effectiveness of immune checkpoint blockade therapy is limited, benefiting only a select group of lung cancer patients [9].

A cornerstone of tumor immunology posits that cytotoxic CD8 + T cells can eliminate tumor cells [10, 11]. Numerous studies corroborate the significant correlation between the presence of cytotoxic CD8 + T cells and enhanced survival rates in lung cancer patients [12]. It has also been established that immune subtypes across various tumors exhibit tissue specificity, each encompassing distinct immune components [13, 14]. Consequently, the ability to accurately identify immune subtypes predictive of an immunotherapeutic response in LUAD patients and to discern the most suitable candidates for immunotherapy would immensely benefit this patient demographic [15–17]. Past research on the immune subtypes of LUAD has predominantly characterized these subtypes using a generalized immune score, focusing solely on the impact of immune infiltration on this score [18–20]. Subsequent studies have highlighted the reversal of CD8 + T cell exhaustion as a pivotal anti-tumor strategy for ICIs [21]. This exhaustion in CD8 + T cells, induced by tumors, is frequently associated with alterations in gene expression [22, 23]. Identifying key genes influencing CD8 + T cell exhaustion, therefore, holds significant potential for enhancing ICI therapy in patients with LUAD [24].

In this study, we conducted analyses on three independent datasets from TCGA and GEO, identifying a set of genes co-expressed with CD8 + T cells. These genes served as markers for consensus clustering within the TCGA and GEO datasets, leading to the delineation of four robust immune subtypes. Systematic analysis further categorized these subtypes into groups with high and low responses to immunotherapy. Through in vitro experiments targeting five pivotal genes associated with CD8 + T cells, we elucidated their roles in the interaction between tumor cells and CD8 + T cells. This research furnishes a scientific basis for enhancing ICI treatments in LUAD.

## Materials and methods

### Data downloading and differentially expressed genes (DEGs) screening

Data comprising 497 tumor and 54 normal samples (in both count and FPKM formats) were retrieved from The Cancer Genome Atlas (TCGA: https://cancergenome.nih.gov/). Additionally, datasets GSE68465 and GSE31210 were acquired from the Gene Expression Omnibus (GEO: https://www.ncbi.nlm.nih.gov/geo/) database. The dataset from the IMvigor210 cohort, which includes numerous cancer patients who have undergone treatment with anti-PD-L1 agents, was obtained from a publicly accessible data package at http://research-pub.gene.com/IMvigor210CoreBiologies/. Differential gene expression analysis between normal and tumor tissues in the TCGA-LUAD cohort was performed utilizing the R package “edgeR”, applying a threshold of |log2 fold-change| > 1 and *p*-value < 0.05 (in count format). Both the ethical consent and informed consent, for research involving human subjects, were granted and duly signed by all participating patients, through the Committees for Ethical Review at Affiliated Hospital of Guangdong Medical University (Approval PJKT2023-156).

### Evaluation of immune cell infiltration

CIBERSORT is a tool designed to estimate the proportions of 22 different immune cell types based on gene expression profiles [25]. It, along with the Tumor Immune Estimation Resource (TIMER) (https://cistrome.shinyapps.io/timer/), was utilized to assess the levels of immune cell infiltration in each LUAD sample [26]. Samples that yielded a *p*-value less than 0.05 were considered to have accurately measured immune cell infiltration fractions as determined by CIBERSORT.

### Construction of weighted co-expressed networks and identification of hub genes related to CD8 + T cells

The R package “WGCNA” facilitated the construction of weighted co-expression networks for differentially expressed genes (DEGs). A soft-threshold power of β = 3 was selected to identify the key module genes. Subsequently, these genes were queried in the STRING database to establish a Protein-Protein Interaction (PPI) network, which was then visualized through Cytoscape software. Hub genes associated with CD8 + T cells were identified by applying the Maximal Clique Centrality (MCC) algorithm within Cytoscape.

### Single-cell sequencing analysis from public databases

SC2diseases (http://easybioai.com/sc2disease/) was a database deriving from a large number of human single-cell sequencing studies [27]. It facilitated the identification of differential genes (logFC > 2) amongst CD8 + exhausted T cells compared to CD8 + non-exhausted T cells in NSCLC patients, drawn from single-cell sequencing research encompassing 3 squamous cell carcinomas and 11 adenocarcinomas. Furthermore, an online tool developed from this research (http://lung.cancer-pku.cn/) enabled the visualization of specific gene expression levels in CD8 + T cells within lung adenocarcinoma.

### Identification of immune subtypes for LUAD

To identify LUAD subtypes distinguished by varying immune characteristics, we utilized the R package “ConsensusClusterPlus” to categorize LUAD patients into subtypes according to genes associated with CD8 + T cell modules. We then conducted principal component analysis (PCA) using R software to evaluate the classification accuracy.

### Analysis of immune components of LUAD immune subtypes

R package “GSVA” was used to perform single-sample gene set enrichment analysis (ssGSEA) for samples of LUAD based on 29 immune gene sets. The immune score, stromal score and tumor purity were calculated by using R package “ESTIMATE”.

### Prediction of immunotherapy response and IC_50_ of chemotherapeutic drugs in LUAD immune subtypes

The “Tumor Immune Dysfunction and Exclusion (TIDE)” method calculates T cell dysfunction and T cell exclusion scores to predict immunotherapy outcomes. A lower TIDE score signifies improved immunotherapy effectiveness. Upon determining the TIDE scores, we categorized LUAD patients with TIDE values greater than 0 as non-responders and those with values less than 0 as responders. The “Immunophenoscore (IPS)” analysis evaluates patient immunogenicity based on effector cells, immunosuppressive cells, MHC molecules, and immunomodulators. An increase in IPS score correlates with enhanced immunogenicity, suggesting a potential for better immunotherapy response. We retrieved IPS scores for LUAD patients from The Cancer Immunome Atlas (TCIA) (https://tcia.at/home). Additionally, the IC50 values for cisplatin, gefitinib, and gemcitabine in each sample were computed using the “pRRophetic” R package.

### Dimension reduction analysis and generation of immunotherapy response signatures for anti-PD-L1 treatment effect prediction

The R package “limma” facilitated the identification of differentially expressed genes (DEGs) between groups with high and low responses to immunotherapy within the TCGA cohort, applying a threshold of | log2 fold-change | > 1 and *p* < 0.05 (in FPKM format). Subsequently, KEGG and GO enrichment analyses of these DEGs were performed using Metascape (http://metascape.org/gp/index.html). Principal component analysis (PCA) utilized the DEGs from both response groups in the TCGA cohort as biomarkers within the IMvigor210 cohort. Principal Component 1 (PC1) was extracted to serve as the immunotherapy response score (ITRscore). Using the optimum cut-off value, the IMvigor210 cohort was stratified into groups with high and low ITRscores to conduct an overall survival analysis. This was done to evaluate the difference in anti-PD-L1 treatment response between these groups. The “survminer” R package determined the optimal cut-off value.

### Somatic mutation analysis and gene set variation analysis (GSVA) of high and low immunotherapy response groups

The MAF files containing somatic mutation data for LUAD were downloaded from TCGA and visualized with the R package “maftools”. Enrichment scores for KEGG pathways and GO biological processes were calculated using the R package “GSVA”. Gene sets corresponding to KEGG pathways and GO biological processes were obtained from the Gene Set Enrichment Analysis (GSEA) database (https://www.gsea-msigdb.org/gsea/index.jsp).

### Cell lines and cell culture

The human lung adenocarcinoma cell line A549 was obtained from the Cell Bank of the Type Culture Collection of the Chinese Academy of Sciences (Shanghai, China). These cells were maintained in DMEM (HyClone, USA) supplemented with 10% heat-inactivated fetal bovine serum (FBS). CD8 + T cells were purified utilizing the CD8 + T Cell Isolation Kit from (Miltenyi Biotech, Germany). For in vitro assays, A549 and CD8 + T cells were treated with various inhibitors: CXCL9 inhibitor (Seselin, 10µM), FOXP3 inhibitor (Epirubicin, 5µM), CD9 inhibitor (Loncastuximab, 15µM), CTLA4 inhibitor (Zalifrelimab, 10µM), or IFN-γ inhibitor (IFN-γ Antagonist 1, 35µM) for 24 h. Cultures were incubated at 37 °C in a 5% CO2 atmosphere.

### CCK-8 assay

A549 cells and CD8 + T cells were co-cultured in 96-well plates at a density of 1,500 cells per well and subjected to treatment with the inhibitors which were mentioned in cell culture. Twenty-four hours post-treatment, the cells were incubated in fresh medium supplemented with CCK-8 assay reagent at a concentration of 0.5 mg/mL (Sigma, USA). Absorbance was measured at 450 nm one hour after incubation.

### EdU assay

A549 cells and CD8 + T cells were co-cultured and subjected to treatment with the inhibitors which were mentioned in cell culture. Twenty-four hours post-treatment, A549 cells were exposed to 10 µM of EdU (Sigma, USA) and incubated for 2 h. Subsequently, the cells were washed thrice with PBS and blocked for 15 min. Following the blocking step, the cells were stained with a fluorescent dye mixture for 2 h, according to the manufacturer’s instructions. Finally, the cells were washed three times with PBS and then examined under a fluorescence microscope.

### Flow cytometry

A549 cells and CD8 + T cells were co-cultured and treated with specified inhibitors which were mentioned in cell culture. Twenty-four hours post-treatment, the cells were suspended at a concentration of 1 × 10^6 cells/ml in flow staining buffer, which consisted of PBS, 0.5% bovine serum albumin, 2 mM EDTA, and 0.1% sodium azide. Subsequently, they were incubated with either anti-PD-L1 (Abcam, UK) or anti-PD1 (Abcam) antibodies for one hour at 4 °C in darkness. The stained cells were then analyzed using a flow cytometer.

### Real-time PCR

Total RNA was extracted from the specified tissues or cells using Trizol reagent (Invitrogen, USA) and then converted to cDNA in the presence of PrimeScript RT Master Mix (Takara, Japan). Quantitative PCR (qPCR) was performed with SYBR premix Ex TaqII reagent (Takara), using β-Actin as the internal control. The qPCR assays utilized primers for IFN-γ: 5′-TCGGTAACTGACTTGAATGTCCA-3′ (forward) and 5′-TCGCTTCCCTGTTTTAGCTGC-3′ (reverse); and for IL-2: 5′-AACTCCTGT CTTGCATTGCAC-3′ (forward) and 5′-GCTCCAGTTGTAGCTGTGTTT-3′ (reverse).

### Elisa assays

A549 cells and CD8 + T cells were cocultured and subjected to treatment with the inhibitors which were mentioned in cell culture for 24 h. The levels of IFN-γ and IL-2 were quantified using the respective IFN-gamma and IL-2 ELISA kits (Abcam, UK), following the manufacturer’s protocol.

### Statistical analysis

Statistical analyses were performed using R version 4.0.3. Differential gene expression analysis was conducted using the “edgeR” and “limma” R packages. The Wilcoxon test and Kruskal-Wallis test were applied for comparisons between two groups and across four groups, respectively. Kaplan-Meier method facilitated the overall survival analyses, with the log-rank test being utilized for group comparisons. A *p*-value of < 0.05 was considered statistically significant. Data were analyzed using GraphPad Prism version 9.0. Student’s t-test in GraphPad Prism was employed to assess differences between groups, with data presented as the mean ± SD from a minimum of three independent experiments. Statistical significance was assigned at *p < 0.05, **p < 0.01, or ***p < 0.001.

## Results

### The royalblue module was significantly associated with CD8 T + and other multiple immune cells

**Fig. 1:**
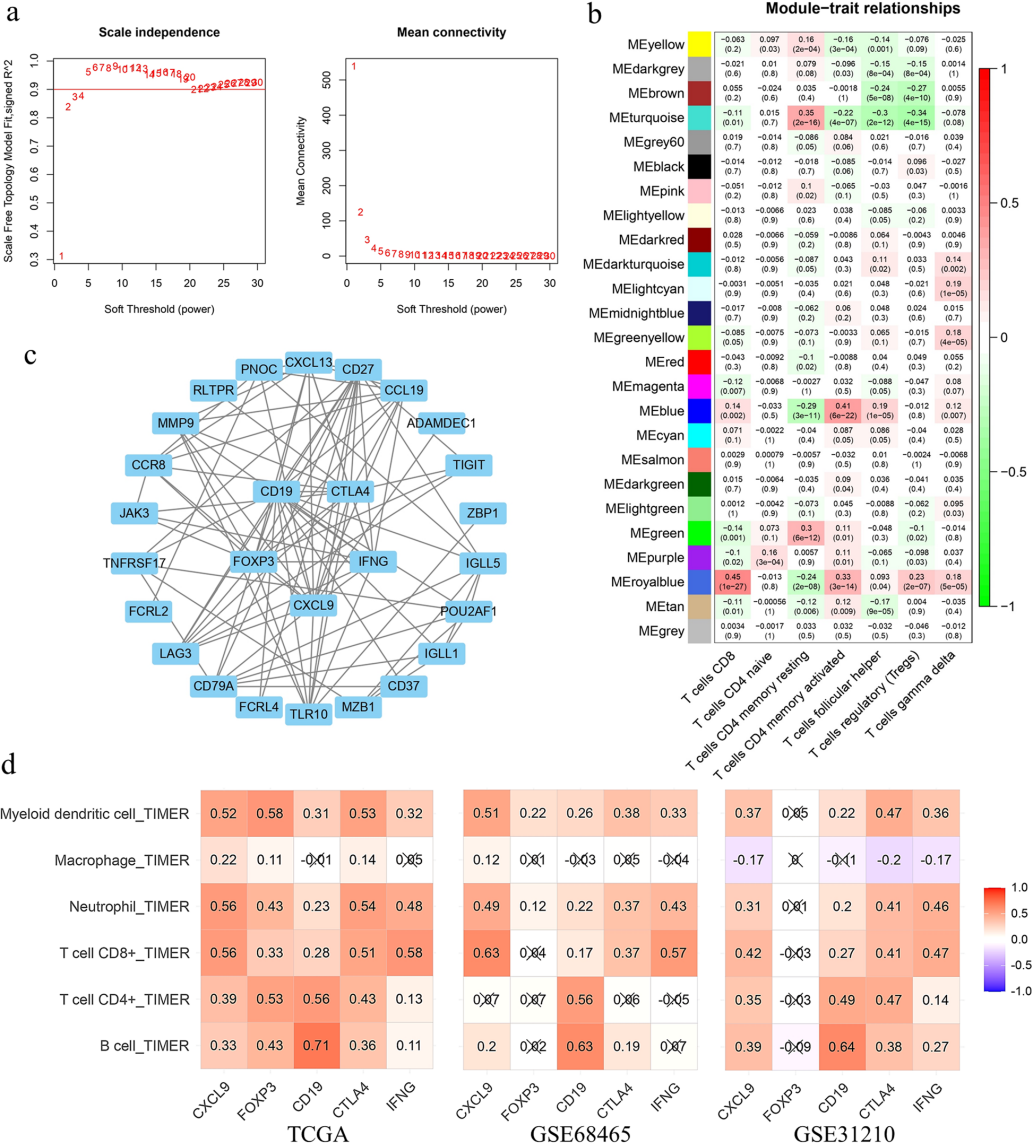
Screening of CD8 + T cell-related weighted co-expressed gene sets in TCGA-LUAD cohort. (**a**) Soft threshold power analysis was used to obtain the scale free fitting index of network topology. (**b**) The correlation between co-expression modules constructed by WGCNA and T cells. The royalblue module had the highest correlation with CD8 + T cells. (**c**) PPI network of royalblue module genes, the top5 hub genes were in the center. (**d**) The correlation between bub genes and B cell, CD4 + T cell, CD8 + T cell, Neutrophil, Macrophage and Myeloid dendritic cell in TCGA, GSE68465 and GSE31210 cohorts

In the TCGA cohort study, 5072 differentially expressed genes (DEGs) were identified with | log2(fold change) |> 1 and a significance level of *p* < 0.05. Using an optimal soft threshold of β = 3, as shown in Figs. 1a and 25 co-expression modules were ultimately identified. The proportions of 22 immune cell types were quantified using CIBERSORT, and 447 tumor samples were selected based on meeting our criteria (*P* < 0.05). Through WGCNA, we explored the correlation between these modules and seven T cell subtypes. Notably, the royalblue module exhibited a significant correlation with CD8 + T cells (Fig. 1b). In the royalblue module, 45 co-expressed genes were identified, which will serve as clustering signatures in subsequent analyses (Supplementary Table 1). Gene Significance was determined by the absolute value of the correlation coefficient between gene expression levels and the proportion of T cells. The PPI network for the genes within the royalblue module was constructed using STRING and visualized through Cytoscape software. The hub genes—CXCL9, FOXP3, CD19, CTLA4, and IFNG—were identified by employing the Maximal Clique Centrality (MCC) algorithm within Cytoscape (Fig. 1c). The infiltration levels of B cells, CD8 + T cells, CD4 + T cells, neutrophils, macrophages, and myeloid dendritic cells were assessed using TIMER. The hub genes (CXCL9, FOXP3, CD19, CTLA4, and IFNG) exhibited a positive correlation with these six types of immune cells in both TCGA and GEO cohorts (Fig. 1d). Additionally, 90 differentially expressed genes, upregulated in CD8 + exhausted T cells, were identified by comparing CD8 + exhausted T cells with CD8 + non-exhausted T cells via SC2diseases (Supplementary Table 2). Among the identified genes, CTLA4, CXCL13, IFNG, LAG3, SIRPG, TIGIT and CD27 coincide with those in the royal blue module. The expression profiles of these seven genes across CD8 + T cell subtypes are depicted in Supplementary Fig. 1. In summary, these findings indicate that the hub genes (CXCL9, FOXP3, CD19, CTLA4, and IFNG) along with their co-expressed counterparts, are significantly associated with CD8 + T cells.

### Immune subtyping of LUAD based on genes of royalblue module related to CD8 + T cells

**Fig. 2:**
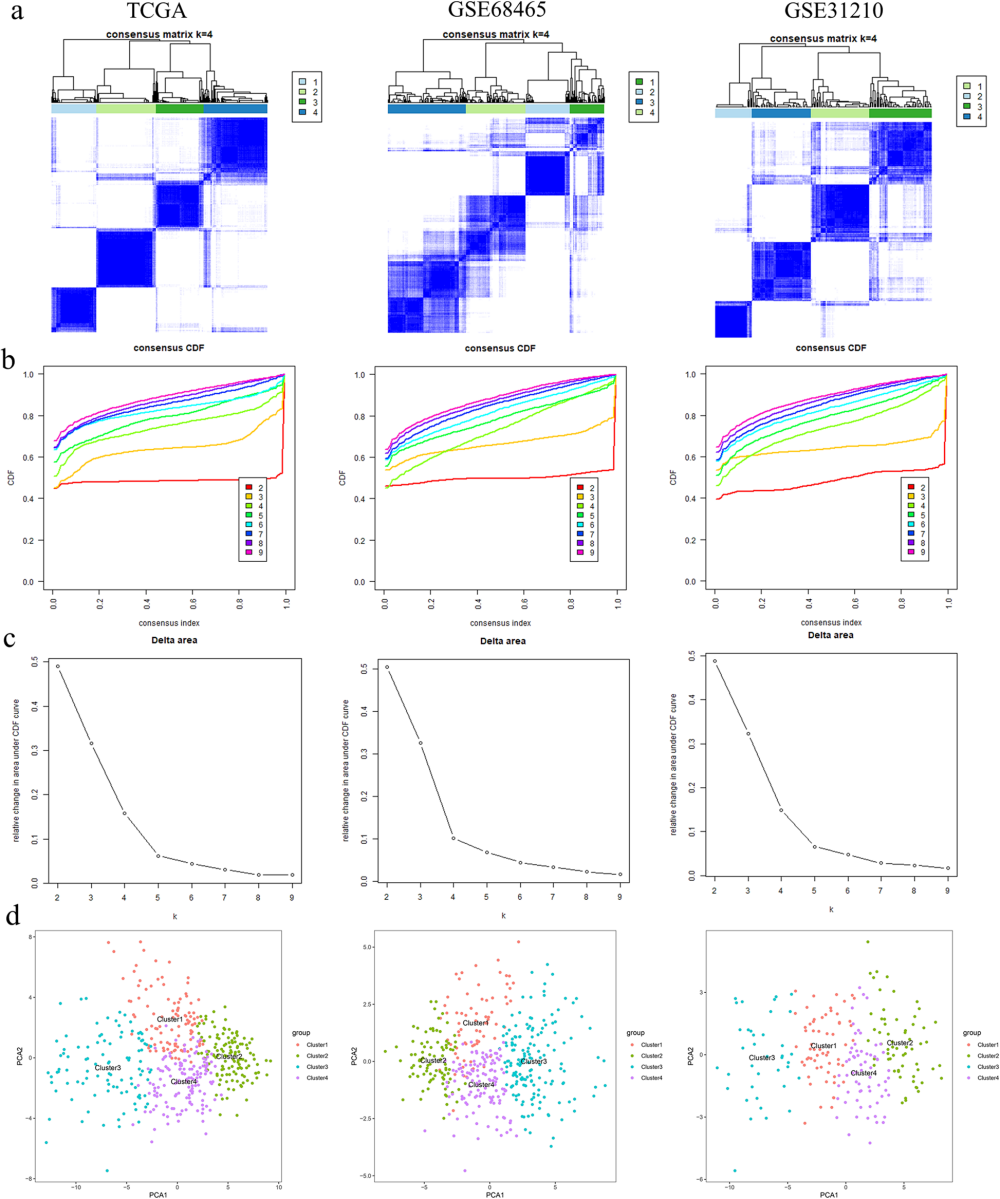
Consensus clustering of three independent cohorts. (**a**) Consensus clustering matrix of three independent cohorts (TCGA, GSE68465 and GSE31210) when k = 4. (**b**) Cumulative distribution function (CDF) of consensus clustering of three independent cohorts (TCGA, GSE68465 and GSE31210) for k = 2–9. (**c**) Relative change of area under CDF curve of three cohorts (TCGA, GSE68465 and GSE31210) for k = 2–9. (**d**) Principal component analysis (PCA) of three cohorts (TCGA, GSE68465 and GSE31210) based on CD8^+^ T cell-related co-expressed genes

Utilizing the genes from the royal blue module, the analyses classified patients from the TCGA-LUAD, GSE68465, and GSE31210 cohorts into four distinct subtypes (TCGA-LUAD: C1: 106, C2: 131, C3: 100, C4: 140; GSE68465: C1: 70, C2: 90, C3: 160, C4: 122; GSE31210: C1: 61, C2: 62, C3: 38, C4: 65) (Fig. 2a-c). PCA revealed clear differentiation among these four immune subtypes of LUAD across the cohorts (Fig. 2d).

### The immune landscapes of immune subtypes of LUAD

**Fig. 3:**
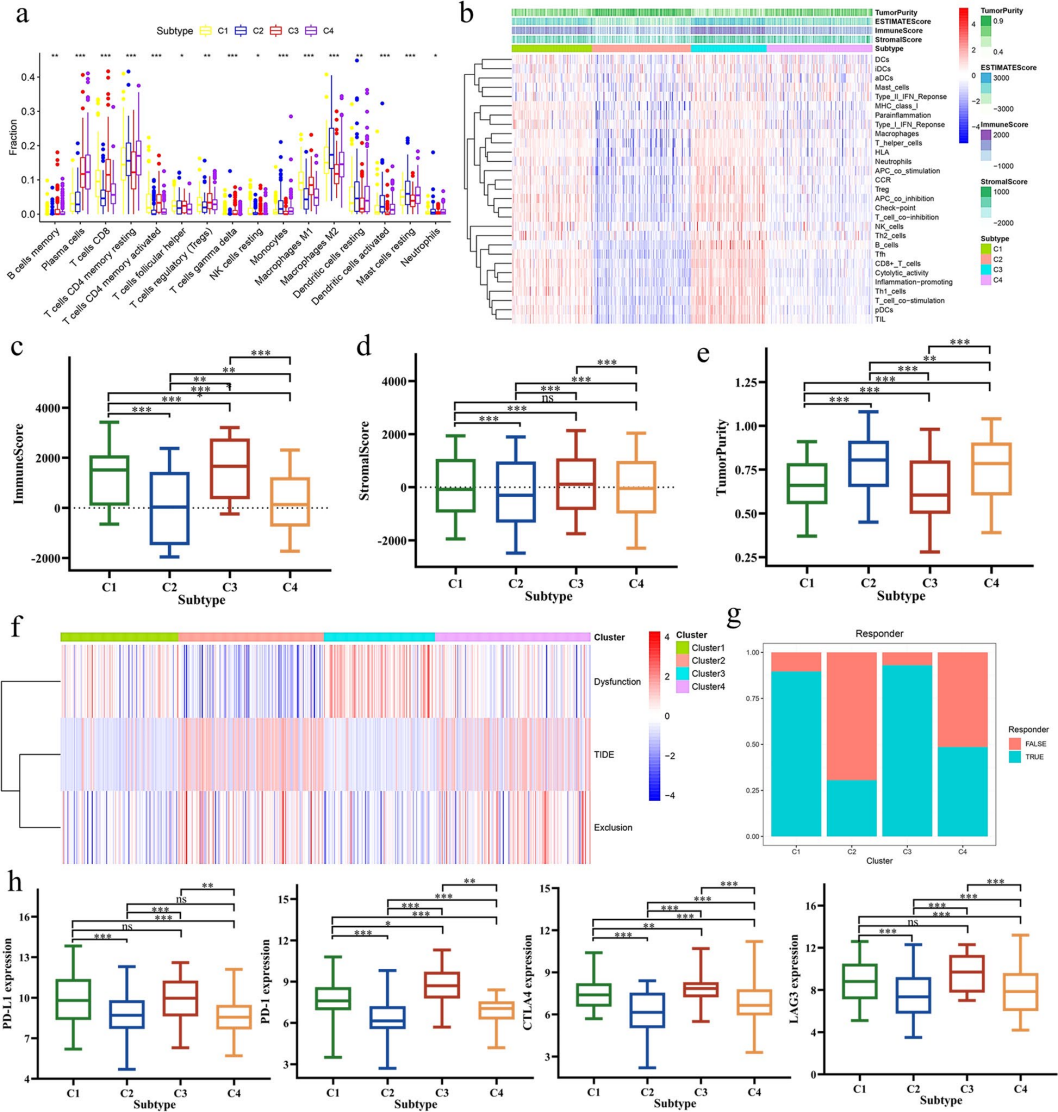
Immune landscape analysis and immunotherapy response analysis of LUAD immune subtypes (**a**) The four immune subtypes of the TCGA cohort showed an almost consistent immune cell infiltration landscape. (**b**) Heatmap of 29 immune gene sets for four immune subtypes in TCGA cohort. (**c**) Immune score of four immune subtypes in TCGA cohort. (**d**) Stromal score of four immune subtypes in TCGA cohort. (**e**) Tumor purity of four immune subtypes in TCGA cohort. (**f**) Heatmap of TIDE score, exclusion score and dysfunction score of four immune subtypes. (**g**) Percentages of true responder of immunotherapy in four immune subtypes (C1: 90%, C2: 31%, C3: 93%, C4: 49%). (**h**) Expression levels of PD-1, PD-L1, CTLA4 and LAG3 in four immune subtypes of TCGA cohort

The composition of immune cells within the immune subtypes, as depicted in Fig. 3a and Supplementary Fig. 2a, shows that CD8 + T cells and M1 macrophages are significantly more abundant in subtypes C1 and C3 compared to C2 and C4. In the TCGA-LUAD cohort, 29 immune gene sets were notably enriched in C1 and C3 (Fig. 3b). A comparison of stromal scores, immune scores, and tumor purity across the four subtypes revealed that both the stromal and immune scores were markedly higher in C1 and C3 than in C2 and C4 (Fig. 3c and d). Conversely, tumor purity was found to be higher in C2 and C4 (Fig. 3e). The findings indicated that subtypes C1 and C3 exhibited more robust immune component profiles than those observed in subtypes C2 and C4. To confirm the stability of these immune subtypes, identical analyses were conducted on the GSE68465 and GSE31210 cohorts. The outcomes of these additional analyses paralleled the trend noted in the TCGA-LUAD cohort, (Supplementary Fig. 2b-2d). Consequently, these findings underscore the reliability and stability of the classification method, confirming the existence of these immune subtypes in LUAD.

### Prediction of immunotherapy response and chemotherapy sensitivity in immune subtypes of LUAD

**Fig. 4:**
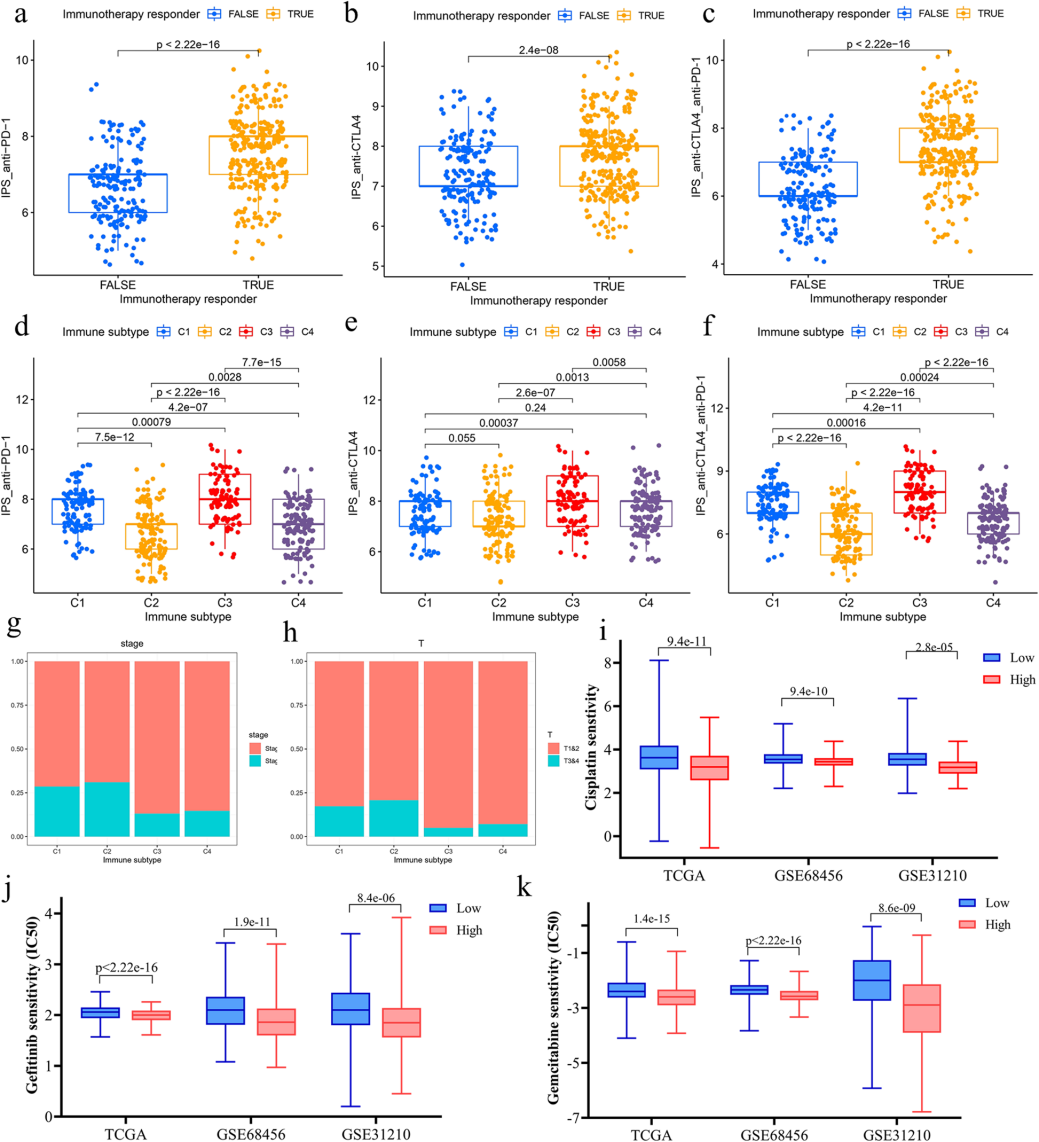
Immunotherapy response and chemotherapy sensitivity analysis of LUAD immune subtypes (a-b) The IPS anti-PD-1 (**a**) and anti-CTLA4 (**b**) scores of immunotherapeutic true responders were significantly higher than false responders. (**c**) The combination therapy of anti-PD-1 and anti-CTLA4 had a higher response in immunotherapeutic true responders. (**d**) C1 and C3 had higher response to anti-PD-1 treatment. (**e**) Only C3 had a high response to anti-CTLA4 treatment. (**f**) C1 and C3 had high response to combination therapy of anti-PD-1 and anti-CTLA4. (g-h) C1 and C2 had a higher proportion of patients with high tumor stages III&IV (**g**) and T3&4 (**h**) than C3 and C4. (i-k) The comparison of IC50 of cisplatin, gefitinib and gemcitabine in high and low immunotherapy response groups in TCGA (**i**), GSE68465 (**j**) and GSE31210 (**k**) cohorts

In this study, we evaluated various biomarkers including TIDE scores, T cell exclusion scores, T cell dysfunction scores, immune checkpoint gene expression levels, and IPS to predict the response to immunotherapy across four immune subtypes. The TIDE algorithm was utilized to compute the TIDE, T cell exclusion, and T cell dysfunction scores for these subtypes. Within the TCGA cohort, subtypes C1 and C3 exhibited higher T cell dysfunction scores and lower TIDE scores, while subtypes C2 and C4 demonstrated lower T cell dysfunction scores and higher TIDE scores (Fig. 3f). The proportion of true immunotherapy responders decreased in the order of C3 (93%) > C1 (90%) > C4 (49%) > C2 (31%) (Fig. 3g). Regarding immune checkpoints, the expression levels of key genes (PD-L1, PD-1, CTLA4, and LAG3) were elevated in subtypes C1 and C3 compared to C2 and C4 across the TCGA-LUAD, GSE68465, and GSE31210 cohorts (Fig. 3h and Supplementary Fig. 3a-b). In the TCGA cohort, true responders to immunotherapy had higher scores in IPS_anti-PD-1, IPS_anti-CTLA4, and IPS_combination of anti-CTLA4 and anti-PD-1 (Fig. 4a-c). In analyzing the immune subtypes, it was notably discovered that, despite both subtypes C1 and C3 having substantial proportions of true immunotherapy responders (90% in C1 and 93% in C3), their responses to specific treatments differed. Subtype C1 exhibited a diminished response to anti-CTLA4 therapy but showed enhanced responsiveness to both anti-PD-1 treatment and the combined regimen of anti-PD-1 and anti-CTLA4. Conversely, subtype C3 demonstrated high efficacy in response to anti-CTLA4, anti-PD-1, and the combination therapy of anti-PD-1 and anti-CTLA4 (Fig. 4d-f). As a result of these findings, subtypes C1 and C3 were categorized as having a high likelihood of responding to immunotherapy, whereas subtypes C2 and C4 were classified under a low response category. An additional intriguing observation was the disparity in the distribution of patients with clinical stages III&IV and T3&4 tumors within these groups. Specifically, within the high response group, subtype C1 contained a greater percentage of patients in these advanced stages compared to C3. Similarly, within the low response group, subtype C2 exhibited a higher incidence of patients in stages III&IV and T3&4 (Fig. 4g and h). Subsequently, the IC50 of cisplatin, gefitinib, and gemcitabine were evaluated across the high and low immunotherapy response groups. To delve deeper into the variances in chemotherapy sensitivity among these groups, the IC50 values for cisplatin, gefitinib, and gemcitabine were assessed within both high and low immunotherapy response cohorts across the TCGA, GSE68465, and GSE31210 datasets. Our findings revealed that the group exhibiting higher immunotherapy responsiveness demonstrated increased sensitivity to cisplatin, gefitinib, and gemcitabine (Fig. 4i-k). Consequently, these observations support the classification of C1 and C3 as indicative of a high immune response, and C2 and C4, of a low immune response, with the former group showcasing a heightened susceptibility to chemotherapy.

### The landscapes of mutation and GSVA enrichment among high and low immunotherapy response groups

**Fig. 5:**
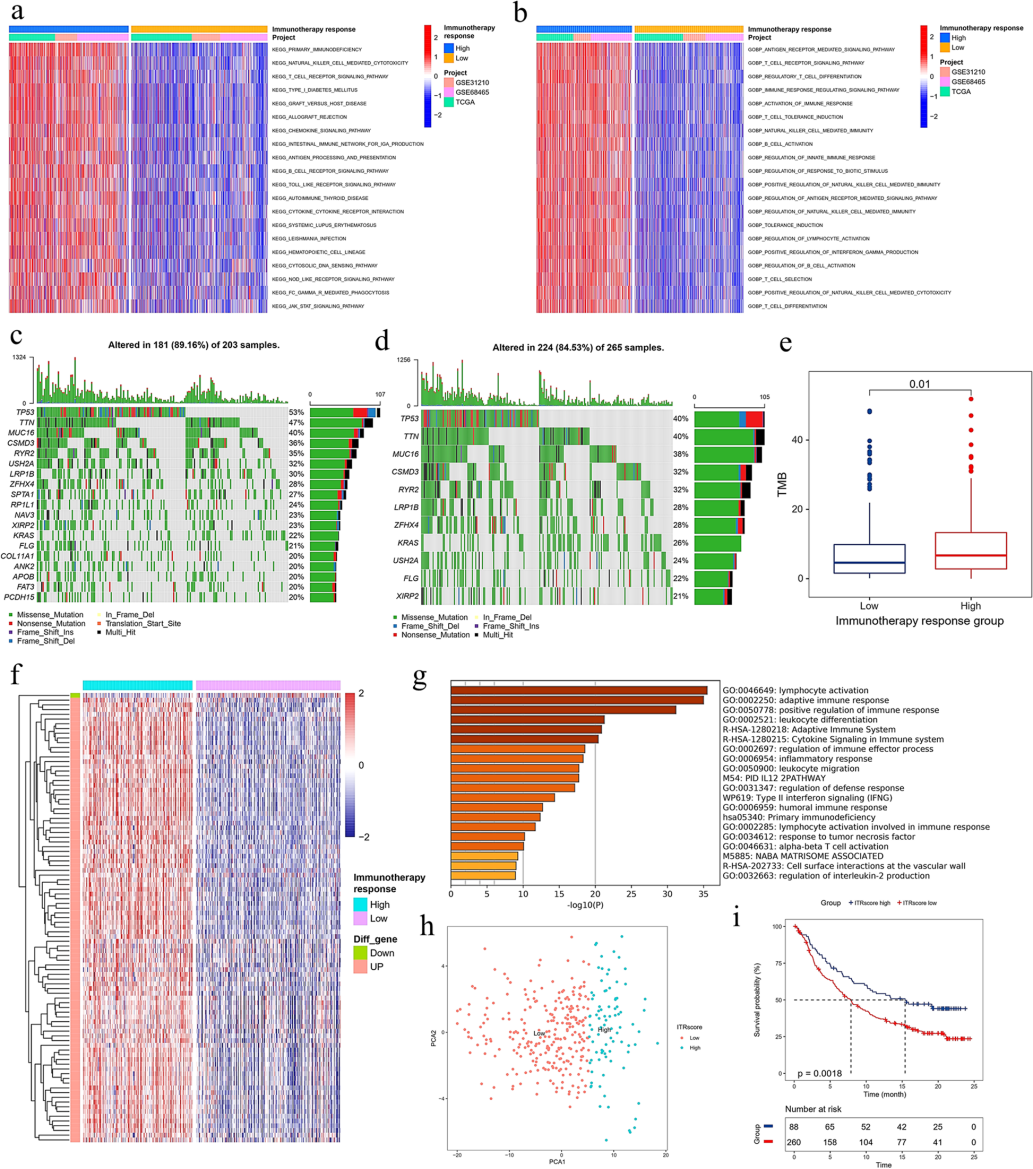
Analysis of immune-related pathways and biological processes in high and low immune response groups and evaluation of anti-PD-L1 therapy. (a) Multiple immune-related pathways were activated in the high immunotherapy response group. (**b**) Various immune-related biological processes were more active in high immunotherapy response group. (**c**) Waterfall plot of mutated genes with mutation frequency greater than 20% of high immunotherapy response group. (**d**) Waterfall plot of mutated genes with mutation frequency greater than 20% of low immunotherapy response group. (**e**) The high immunotherapy response group had higher TMB. (**f**) Heatmap of DEGs of high and low immunotherapy response groups. (**g**) Function annotation of DEGs. (**h**) PCA analysis of DEGs. PC1 was extracted to serve as ITRscore. (**i**) Kaplan-Meier plot of IMvigor210 cohort. The IMvigor210 cohort was divided into high and low ITRscore group according to the best cut-off value of ITRscore.

To deepen our understanding of the disparities between high and low immunotherapy response groups, further analyses were conducted focusing on pathways and biological processes across the TCGA, GSE31210, and GSE68465 cohorts. The group exhibiting a high response to immunotherapy demonstrated activation across numerous immune-related pathways, including primary immunodeficiency, cytotoxicity mediated by natural killer cells, T cell receptor signaling, allograft rejection, antigen processing and presentation, and B cell receptor signaling, among others (Fig. 5a). Similarly, this group showed pronounced activity in various immune-related biological processes, such as differentiation of regulatory T cells, activation of the immune response, induction of T cell tolerance, activation of B cells, and regulation of the innate immune response (Fig. 5b). In the TCGA cohort, genes exhibiting a mutation frequency exceeding 20% were identified in both high and low immunotherapy response groups (Fig. 5c and d). The high response group contained 19 genes with mutation frequencies above 20%, whereas the low response group comprised only 11. Generally, the same genes exhibited higher mutation frequencies in the high response group. Furthermore, the tumor mutational burden was elevated in the high response group (Fig. 5e). These findings suggest that the group with a high response to immunotherapy demonstrated greater immunogenicity.

### The ITRscore predicts immunotherapeutic benefits of anti-PD-L1

To investigate the molecular features distinguishing high from low immunotherapy response groups, a differential gene analysis was performed within the TCGA cohort comparing these groups. Applying a threshold of absolute log2 fold-change > 1 and *p* < 0.05, 95 DEGs were identified, comprising 94 upregulated and 1 downregulated gene (Fig. 5f). These genes were implicated in diverse immune-related functions including lymphocyte activation, adaptive immune response, and positive regulation of immune responses, among others (Fig. 5g). Utilizing these DEGs, the PCA algorithm was employed to calculate the ITRscore, stratifying patients who underwent anti-PD-L1 therapy in the IMvigor210 cohort into high ITRscore (*n* = 88) and low ITRscore (*n* = 260) groups (Fig. 5h). Notably, patients in the high ITRscore group experienced significantly longer overall survival compared to those in the low ITRscore group within the IMvigor210 cohort (Fig. 5i), indicating that patients sharing the molecular signature of the high immunotherapy response group benefited more from anti-PD-L1 treatment.

### Inhibition of these five hub genes can increase CD8^+^T cell activity and inhibit tumor growth

**Fig. 6:**
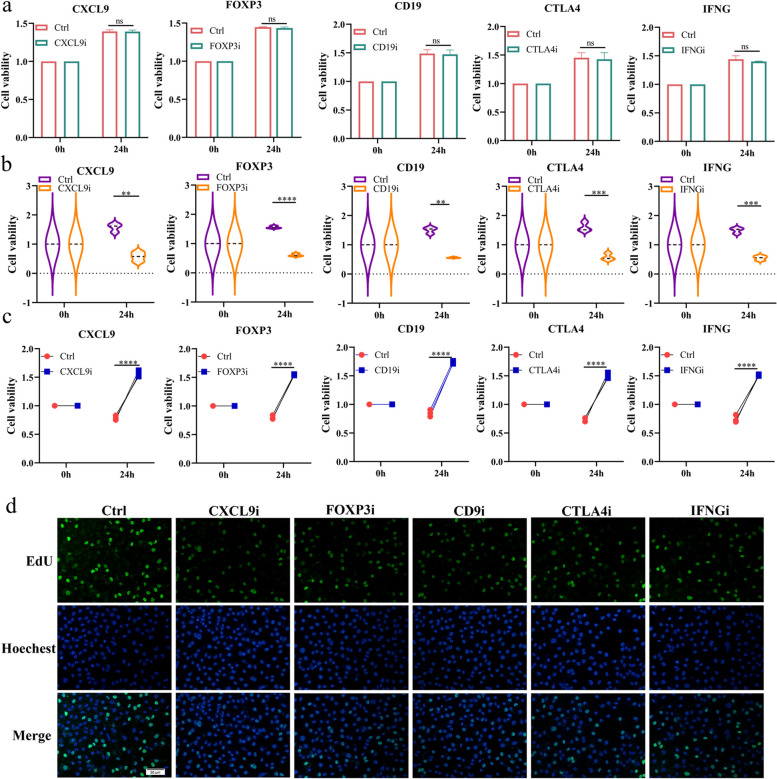
Inhibition of these five hub genes on the function of LUAD cells and CD8^+^T cells. (**a**) A549 cells were exposed to CXCL9i (Seselin), FOXP3i (Epirubicin), CD9i (loncastuximab), CTLA4i (Zalifrelimab), or IFNGi (IFN-γ Antagonist 1) for 24 h, and then CCK8 assay was used to detect the changes in cell activity of A549. (**b**) A549 cells were co-cultured with CD8^+^ T cells and exposed to inhibitors of these five hub genes for 24 h, respectively. After that, the cell viability of A549 cells was examined by CCK8 assay. (**c**) Cells were treated as in (**b**). After treatment, the cell viability of CD8^+^ T cells was examined by CCK8 assay. (**d**) A549 cells were co-cultured with CD8^+^ T cells and exposed to inhibitors of these five hub genes for 24 h, respectively. After that, the proliferation ability of A549 cells was examined by EdU assay

Our analysis has revealed a close association between the genes CXCL9, FOXP3, CD9, CTLA4, IFNG, and CD8 + T cells. To investigate the functions of these genes, we initially treated lung adenocarcinoma A549 cells with specific inhibitors: CXCL9i (Seselin), FOXP3i (Epirubicin), CD9i (Loncastuximab), CTLA4i (Zalifrelimab), and IFNGi (IFN-γ Antagonist 1). Unexpectedly, these inhibitors did not significantly affect the viability of the A549 cells in isolation (Fig. 6a). Conversely, in co-culture with CD8 + T cells, a marked reduction in A549 cell viability was observed upon treatment with any of the five inhibitors (Supplementary Fig. 4a and Fig. 6b). Furthermore, the viability of tumor-suppressive CD8 + T cells improved following exposure to these inhibitors (Fig. 6c). Subsequent co-culture experiments exposing A549 cells and CD8 + T cells to the inhibitors confirmed that all five could indeed reduce A549 cell proliferation, as demonstrated by EdU proliferation assays (Fig. 6d and Supplementary Fig. 4b). These findings imply that inhibiting these pivotal genes enhances CD8 + T cell viability and their suppressive effects on lung adenocarcinoma cells.

### Inhibition of these five hub genes can remove tumor immunosuppression of CD8^+^ T cells

**Fig. 7:**
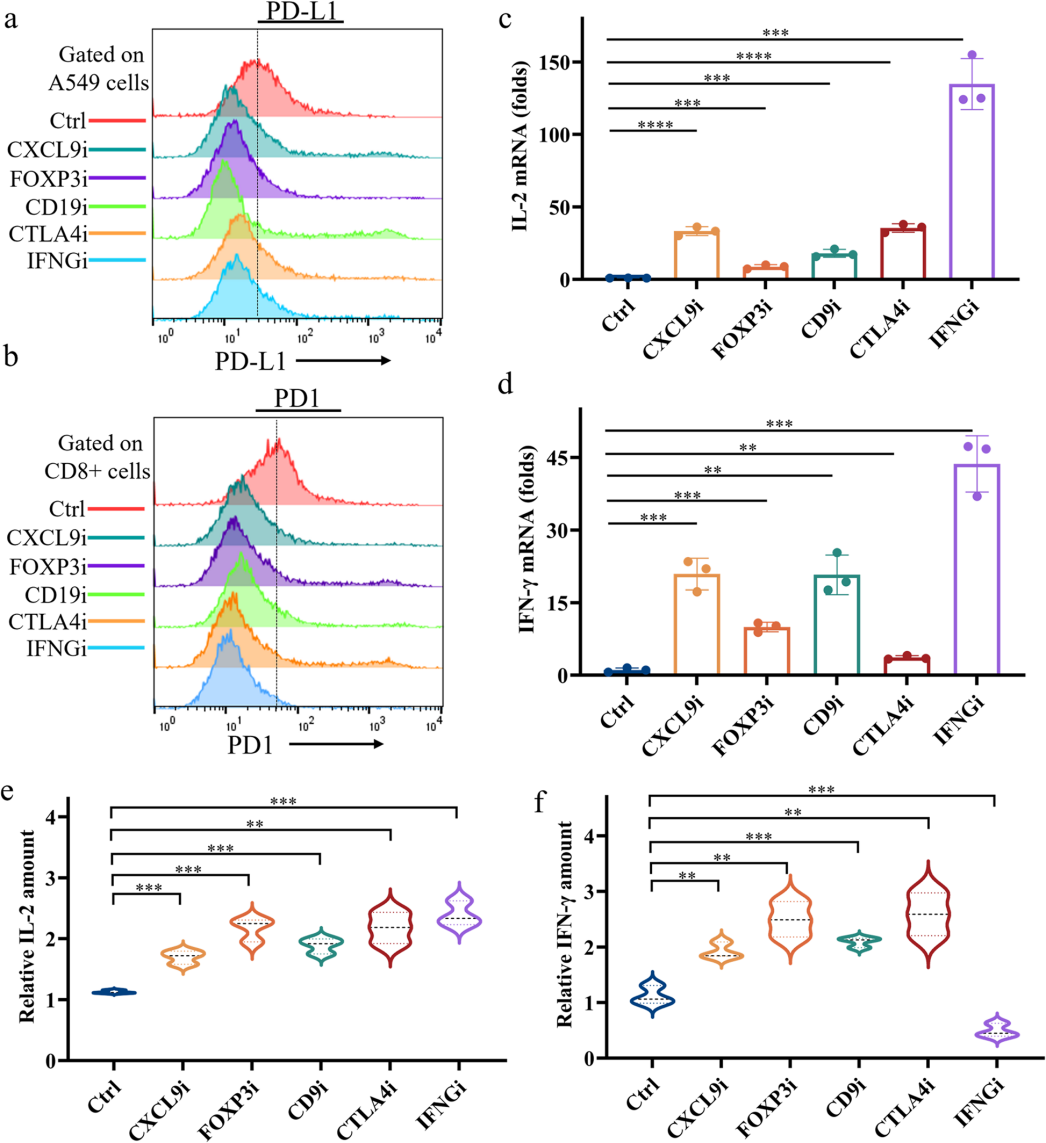
Inhibition of these five hub genes can remove tumor immunosuppression of CD8^+^T cells. (a-b) A549 cells were co-cultured with CD8^+^ T cells and exposed to inhibitors of these five hub genes for 24 h, respectively. After treatment, PD-L1 expression levels on A549 cells (**a**) or PD1 expression levels on CD8^+^ T cells (**b**) were measured by flow cytometry. (**c-d**) Cells were treated as in (**b**). After treatment, mRNA levels of IL-2 (**c**) and IFN-γ (**d**) in CD8^+^ T cells were detected by qPCR assay. (**e-f**) Cells were treated as in (**b**). After treatment, the levels of cytokines IL-2 (**e**) and IFN-γ (**f**) secreted by CD8^+^ T cells were detected by Elisa assay

To further explore the impact of five pivotal genes on CD8 + T cells, we co-cultured A549 cells with CD8 + T cells and treated them with inhibitors targeting these genes. We assessed the expression of PD-L1 on A549 cells and PD1 on CD8 + T cells using flow cytometry. The findings revealed a reduction in PD-L1 levels on A549 cells and a decline in PD1 expression on CD8 + T cells (Fig. 7a and b). Moreover, we quantified the mRNA levels of IL-2 and IFN-γ, cytokines known to influence CD8 + T cell cytotoxicity, through qPCR. The results indicated that inhibitors of the five targeted genes elevated the mRNA levels of IL-2 and IFN-γ (Fig. 7c and d). Furthermore, the secretion of IL-2 by CD8 + T cells was increased in the presence of these inhibitors (Fig. 7e). Conversely, while inhibitors of CXCL9, FOXP3, CD9, and CTLA4 boosted the secretion of IFN-γ by CD8 + T cells, inhibition of IFNG led to a reduction in IFN-γ secretion (Fig. 7f). This outcome is attributable to the role of IFNG in encoding the IFN-γ protein, thereby explaining why blocking IFNG diminishes IFN-γ protein levels. Collectively, our results demonstrate that inhibiting these five central genes may alleviate the immunosuppressive effects of lung adenocarcinoma cells on CD8 + T cells and enhance their cytotoxic capabilities.

## Discussion

Immunotherapy has made significant strides in treating certain LUAD patients. Nevertheless, the tumor microenvironment’s heterogeneity limits its efficacy to only a subset of these patients [28]. Thus, within the framework of precision medicine, identifying LUAD patients who would benefit from immunotherapy is crucial, as is the formulation of tailored mono-therapy and combination treatment strategies [12, 29]. Our study identified 45 DEGs co-expressed in both normal and LUAD tissues, which are associated with the presence of tumor-infiltrating CD8 + T cells. Within this group, five hub genes (CXCL9, FOXP3, CD19, CTLA4, and IFNG) were identified as playing a pivotal role in regulation. Subsequent analyses showed seven of these co-expressed genes to be highly expressed in exhausted CD8 + T cells within lung adenocarcinoma. Utilizing these genes, we classified the TCGA-LUAD cohort into four immune subtypes and analyzed the heterogeneity within these subtypes. We then validated the existence of these immune subtypes by examining the GSE68465 and GSE31210 cohorts. Through a comprehensive analysis of LUAD immune subtypes, we characterized C1 and C3 as indicative of a high response to immunotherapy, and C2 and C4 as indicative of a low response. Regarding chemotherapy sensitivity, the subtypes associated with a high immunotherapy response exhibited significantly reduced IC50 values for cisplatin, gefitinib, and gemcitabine, suggesting enhanced susceptibility. Overall, our findings suggest that the C1 and C3 immune subtypes of LUAD may confer superior clinical outcomes through immunotherapy, chemotherapy, or their combination.

Numerous studies have substantiated the pivotal role of immune-related genes in determining the clinical prognosis of cancer patients and their response to chemotherapy and immunotherapy [30, 31]. In our research, we primarily concentrated on genes co-expressed with CD8 + T cells, employing the MCC algorithm provided by Cytoscape to identify the five central hub genes within this co-expressed set. CTLA4 was found to inhibit T cell activation and proliferation, thereby diminishing tumor immunity [32]. Additionally, FOXP3-expressing Treg cells were observed to suppress both the proliferation and functionality of adjacent T cells [33]. An increase in the expression of the chemokine ligand CXCL9 has been associated with enhanced infiltration of CD8 + T cells in solid tumors [34]. IFNG has been noted for its role in bolstering immune function, albeit it also induces T cell exhaustion via PD-L1, thus constraining both adaptive and innate immunity [35]. Lastly, CD19 serves as an antigen present on the surface of B lymphocytes [36]. In the TCGA and GEO cohorts, we observed a positive correlation between the expression levels of five pivotal hub genes and the infiltration of various immune cells, such as B cells, CD8 + T cells, CD4 + T cells, neutrophils, macrophages, and myeloid dendritic cells. By implementing WGCNA to identify co-expressed genes associated with CD8 + T cells, we effectively discriminated between LUAD immune subtypes exhibiting distinct immune characteristics. Utilizing these WGCNA-derived signatures for clustering facilitated the classification of LUAD patients into three distinct groups across three independent cohorts, yielding stable and concordant outcomes. Notably, subtypes C1 and C3 demonstrated a richer immune composition and exhibited a higher degree of CD8 + T cell exhaustion, alongside a superior proportion of authentic immunotherapy responders (C3: 93%, C1: 90%, compared to C4: 49%, and C2: 31%). Besides, C1 showing an enhanced response to anti-PD-1 therapy and its combination with anti-CTLA4 therapy. Similarly, C3 demonstrated significant responsiveness to both anti-CTLA4 and anti-PD-1 treatments, including their combined use. Additionally, both C1 and C3 exhibited increased sensitivity to cisplatin, gefitinib, and gemcitabine, suggesting that a combination of immunotherapy and chemotherapy could yield substantial clinical benefits for patients classified within these subtypes. Consequently, C1 and C3 were categorized as having a high immunotherapy response, in contrast to C2 and C4, which were classified as having a low response. Enrichment analysis revealed that the high-response groups were characterized by the activation of multiple immune-related pathways. Furthermore, differential gene analysis showed that genes upregulated in the high-response group were predominantly associated with immune-related functions. Notably, the frequency of gene mutations and tumor mutation burdens were significantly higher in the high-response group compared to the low-response group, indicating increased immunogenicity, which likely contributes to their heightened sensitivity to immunotherapy.

To investigate the roles of five hub genes, in vitro assays were performed where inhibitors specific to these genes were introduced to A549 cells in monoculture. Contrary to expectations, the addition of these inhibitors failed to significantly suppress the proliferation of the tumor cells, suggesting that these hub genes may not directly influence tumor cell regulation. Conversely, in co-culture experiments with CD8 + T cells and A549 cells, administration of the same five gene inhibitors not only suppressed A549 cell growth but also enhanced the viability of CD8 + T cells. Furthermore, inhibition of these hub genes attenuated the immunosuppressive effects exerted by the tumor cells on CD8 + T cells and decreased PD1 expression on the latter. This was accompanied by an increase in CD8 + T cell cytotoxicity and elevated levels of the cytokines IFN-γ and IL-2. These findings support the hypothesis that the five hub genes facilitate tumor immune evasion by modulating interactions between tumor cells and CD8 + T cells. Through analyzing previous research, we hypothesized that CD19 influences the recognition of tumor antigens by CD8 + T cells. Furthermore, the levels of CTLA4 and FOXP3 directly impact the proliferation and activation of these cells [37], while CXCL9 affects their infiltration, and IFNG contributes to their depletion [38, 39]. Collectively, these five pivotal genes modulate the efficacy of CD8 + T cells against LUAD through various mechanisms and levels.

## Conclusions

Consequently, our study identified five hub genes closely associated with CD8 + T cell function, categorized LUAD into four subtypes and two immune response groups, thus laying a theoretical foundation for the personalized treatment of LUAD patients and the advancement of ICIs therapy.

### Supplementary Information

Supplementary Material 1.

## Data Availability

The datasets generated in this study are available from the corresponding author upon reasonable request.

## Abbreviations

LUAD: lung adenocarcinoma
WGCNA: weighted gene co-expression network analysis
STRING: Search Tool for the Retrieval of Interacting Genes
TIDE: Tumor Immune Dysfunction and Exclusion
IPS: Immunophenoscore
NSCLC: Non-small cell lung cancer
SCLC: small cell lung cancer
LUSC: squamous cell carcinoma
ICIs: immune checkpoint inhibitors
GEO: Gene Expression Omnibus
TCGA: The Cancer Genome Atlas
TIMER: Tumor Immune Estimation Resource
DEGs: differentially expressed genes
PPI: Protein-Protein Interaction
MCC: Maximal Clique Centrality
PCA: principal component analysis
ssGSEA: single-sample gene set enrichment analysis
TCIA: The Cancer Immunome Atlas
PC1: Principal Component 1
ITRscore: immunotherapy response score
FBS: fetal bovine serum
qPCR: Quantitative PCR
