# Supplementary material for: Comprehensive analysis and identification of subtypes and hub genes of high immune response in lung adenocarcinoma
Source: BMC Pulm Med. 2024 Jul 4;24:324. doi: 10.1186/s12890-024-03130-6 (PMC11225283; doi:10.1186/s12890-024-03130-6)
Supplement: Supplementary file 1 — Supplementary Material 1. [file 12890_2024_3130_MOESM1_ESM.docx]

**Supplementary table 1. CD8+ T cell-related co-expressed genes from royalblue module.**

| **probes** | **moduleColor** | **GS.T cells CD8** | **p.GS.T cells CD8** |
| --- | --- | --- | --- |
| IGLL1 | royalblue | 0.194836 | 8.80E-06 |
| ALPI | royalblue | 0.129808 | 0.003225 |
| IFNG | royalblue | 0.584826 | 2.15E-48 |
| FCRL4 | royalblue | 0.249322 | 1.04E-08 |
| TTC24 | royalblue | 0.585152 | 1.85E-48 |
| BFSP2 | royalblue | 0.131999 | 0.00274 |
| MEF2B | royalblue | 0.269999 | 5.09E-10 |
| ZNF80 | royalblue | 0.383074 | 2.24E-19 |
| FCRL2 | royalblue | 0.078479 | 0.075747 |
| TNFRSF17 | royalblue | 0.234864 | 7.36E-08 |
| ADAMDEC1 | royalblue | 0.226762 | 2.09E-07 |
| P2RX5 | royalblue | 0.34133 | 1.83E-15 |
| PNOC | royalblue | 0.293215 | 1.25E-11 |
| CCR8 | royalblue | 0.115343 | 0.008927 |
| FCRL5 | royalblue | 0.20034 | 4.81E-06 |
| GPR84 | royalblue | 0.07883 | 0.074442 |
| TLR10 | royalblue | 0.059632 | 0.177487 |
| CD19 | royalblue | 0.262516 | 1.56E-09 |
| MIXL1 | royalblue | 0.213162 | 1.10E-06 |
| PLA2G2D | royalblue | 0.326547 | 3.27E-14 |
| SIRPG | royalblue | 0.678517 | 1.83E-70 |
| KLHDC7B | royalblue | 0.206946 | 2.28E-06 |
| LAG3 | royalblue | 0.535271 | 2.27E-39 |
| MYBPC2 | royalblue | 0.113477 | 0.010104 |
| ZBP1 | royalblue | 0.355962 | 9.05E-17 |
| CARMIL2 | royalblue | 0.244664 | 1.98E-08 |
| CTLA4 | royalblue | 0.315931 | 2.36E-13 |
| POU2AF1 | royalblue | 0.233639 | 8.64E-08 |
| OR2I1P | royalblue | 0.232122 | 1.05E-07 |
| CD79A | royalblue | 0.256341 | 3.85E-09 |
| MZB1 | royalblue | 0.307914 | 9.96E-13 |
| CD27 | royalblue | 0.41808 | 4.02E-23 |
| FOXP3 | royalblue | 0.250933 | 8.31E-09 |
| DERL3 | royalblue | 0.238795 | 4.38E-08 |
| TIGIT | royalblue | 0.521939 | 3.45E-37 |
| CXCL13 | royalblue | 0.28707 | 3.45E-11 |
| IGLL5 | royalblue | 0.282502 | 7.22E-11 |
| IL4I1 | royalblue | 0.191376 | 1.27E-05 |
| CPNE5 | royalblue | 0.261908 | 1.71E-09 |
| HAPLN3 | royalblue | 0.372192 | 2.67E-18 |
| CCL19 | royalblue | 0.225956 | 2.31E-07 |
| JAK3 | royalblue | 0.389953 | 4.45E-20 |
| MMP9 | royalblue | 0.070124 | 0.11266 |
| CXCL9 | royalblue | 0.461911 | 1.78E-28 |
| CD37 | royalblue | 0.20274 | 3.68E-06 |

**Supplementary table 2. Differential genes between CD8+ exhausted T cells (up-regulated) and CD8+ non-exhausted T cells were extracted by using SC2diseases.**

| PaperID | celltype | disease | gene | value | variablename |
| --- | --- | --- | --- | --- | --- |
| 2994204 | CD8 exhausted T cells | Lung cancer | HAVCR2 | 5.88 | logFC |
| 2994204 | CD8 exhausted T cells | Lung cancer | CXCL13 | 5.31 | logFC |
| 2994204 | CD8 exhausted T cells | Lung cancer | CCL3 | 5.02 | logFC |
| 2994204 | CD8 exhausted T cells | Lung cancer | SIRPG | 4.64 | logFC |
| 2994204 | CD8 exhausted T cells | Lung cancer | IFNG | 3.94 | logFC |
| 2994204 | CD8 exhausted T cells | Lung cancer | TIGIT | 3.82 | logFC |
| 2994204 | CD8 exhausted T cells | Lung cancer | GZMB | 3.79 | logFC |
| 2994204 | CD8 exhausted T cells | Lung cancer | PDCD1 | 3.6 | logFC |
| 2994204 | CD8 exhausted T cells | Lung cancer | PARK7 | 3.44 | logFC |
| 2994204 | CD8 exhausted T cells | Lung cancer | TNFRSF9 | 3.43 | logFC |
| 2994204 | CD8 exhausted T cells | Lung cancer | ACP5 | 3.38 | logFC |
| 2994204 | CD8 exhausted T cells | Lung cancer | CTLA4 | 3.29 | logFC |
| 2994204 | CD8 exhausted T cells | Lung cancer | RBPJ | 3.22 | logFC |
| 2994204 | CD8 exhausted T cells | Lung cancer | MIR155 | 3.21 | logFC |
| 2994204 | CD8 exhausted T cells | Lung cancer | CXCR6 | 3.21 | logFC |
| 2994204 | CD8 exhausted T cells | Lung cancer | CD27 | 3.21 | logFC |
| 2994204 | CD8 exhausted T cells | Lung cancer | FKBP1A | 3.16 | logFC |
| 2994204 | CD8 exhausted T cells | Lung cancer | BST2 | 3.11 | logFC |
| 2994204 | CD8 exhausted T cells | Lung cancer | TPI1 | 3.1 | logFC |
| 2994204 | CD8 exhausted T cells | Lung cancer | MIR155HG | 3.06 | logFC |
| 2994204 | CD8 exhausted T cells | Lung cancer | PTTG1 | 2.97 | logFC |
| 2994204 | CD8 exhausted T cells | Lung cancer | CD63 | 2.88 | logFC |
| 2994204 | CD8 exhausted T cells | Lung cancer | SAMSN1 | 2.85 | logFC |
| 2994204 | CD8 exhausted T cells | Lung cancer | RGS1 | 2.84 | logFC |
| 2994204 | CD8 exhausted T cells | Lung cancer | CD27-AS1 | 2.84 | logFC |
| 2994204 | CD8 exhausted T cells | Lung cancer | ITGAE | 2.81 | logFC |
| 2994204 | CD8 exhausted T cells | Lung cancer | MIR4632 | 2.81 | logFC |
| 2994204 | CD8 exhausted T cells | Lung cancer | HLA-DRA | 2.77 | logFC |
| 2994204 | CD8 exhausted T cells | Lung cancer | IGFLR1 | 2.74 | logFC |
| 2994204 | CD8 exhausted T cells | Lung cancer | KRT86 | 2.71 | logFC |
| 2994204 | CD8 exhausted T cells | Lung cancer | ENTPD1 | 2.58 | logFC |
| 2994204 | CD8 exhausted T cells | Lung cancer | DUSP4 | 2.56 | logFC |
| 2994204 | CD8 exhausted T cells | Lung cancer | SIT1 | 2.51 | logFC |
| 2994204 | CD8 exhausted T cells | Lung cancer | TOX | 2.5 | logFC |
| 2994204 | CD8 exhausted T cells | Lung cancer | PHLDA1 | 2.5 | logFC |
| 2994204 | CD8 exhausted T cells | Lung cancer | CCND2 | 2.5 | logFC |
| 2994204 | CD8 exhausted T cells | Lung cancer | GPR25 | 2.47 | logFC |
| 2994204 | CD8 exhausted T cells | Lung cancer | LAYN | 2.47 | logFC |
| 2994204 | CD8 exhausted T cells | Lung cancer | PRDX5 | 2.44 | logFC |
| 2994204 | CD8 exhausted T cells | Lung cancer | SARDH | 2.43 | logFC |
| 2994204 | CD8 exhausted T cells | Lung cancer | FASLG | 2.43 | logFC |
| 2994204 | CD8 exhausted T cells | Lung cancer | MIR3917 | 2.42 | logFC |
| 2994204 | CD8 exhausted T cells | Lung cancer | ANXA5 | 2.4 | logFC |
| 2994204 | CD8 exhausted T cells | Lung cancer | CTSD | 2.4 | logFC |
| 2994204 | CD8 exhausted T cells | Lung cancer | PDIA6 | 2.36 | logFC |
| 2994204 | CD8 exhausted T cells | Lung cancer | RANBP1 | 2.36 | logFC |
| 2994204 | CD8 exhausted T cells | Lung cancer | FKBP1A-SDCBP2 | 2.36 | logFC |
| 2994204 | CD8 exhausted T cells | Lung cancer | COTL1 | 2.35 | logFC |
| 2994204 | CD8 exhausted T cells | Lung cancer | TNFRSF1B | 2.35 | logFC |
| 2994204 | CD8 exhausted T cells | Lung cancer | IDH2 | 2.35 | logFC |
| 2994204 | CD8 exhausted T cells | Lung cancer | CD38 | 2.33 | logFC |
| 2994204 | CD8 exhausted T cells | Lung cancer | CD82 | 2.32 | logFC |
| 2994204 | CD8 exhausted T cells | Lung cancer | LAG3 | 2.32 | logFC |
| 2994204 | CD8 exhausted T cells | Lung cancer | MIR497HG | 2.31 | logFC |
| 2994204 | CD8 exhausted T cells | Lung cancer | APOBEC3C | 2.28 | logFC |
| 2994204 | CD8 exhausted T cells | Lung cancer | ITM2A | 2.26 | logFC |
| 2994204 | CD8 exhausted T cells | Lung cancer | COX5A | 2.25 | logFC |
| 2994204 | CD8 exhausted T cells | Lung cancer | IFI35 | 2.25 | logFC |
| 2994204 | CD8 exhausted T cells | Lung cancer | NDFIP2 | 2.21 | logFC |
| 2994204 | CD8 exhausted T cells | Lung cancer | TNFRSF18 | 2.2 | logFC |
| 2994204 | CD8 exhausted T cells | Lung cancer | KRT81 | 2.19 | logFC |
| 2994204 | CD8 exhausted T cells | Lung cancer | DNPH1 | 2.19 | logFC |
| 2994204 | CD8 exhausted T cells | Lung cancer | RGS2 | 2.19 | logFC |
| 2994204 | CD8 exhausted T cells | Lung cancer | HMGN1 | 2.19 | logFC |
| 2994204 | CD8 exhausted T cells | Lung cancer | DYNLL1 | 2.18 | logFC |
| 2994204 | CD8 exhausted T cells | Lung cancer | SNRPB | 2.18 | logFC |
| 2994204 | CD8 exhausted T cells | Lung cancer | STRA13 | 2.16 | logFC |
| 2994204 | CD8 exhausted T cells | Lung cancer | SYNGR2 | 2.16 | logFC |
| 2994204 | CD8 exhausted T cells | Lung cancer | RAB27A | 2.15 | logFC |
| 2994204 | CD8 exhausted T cells | Lung cancer | PSMC3 | 2.15 | logFC |
| 2994204 | CD8 exhausted T cells | Lung cancer | GALM | 2.12 | logFC |
| 2994204 | CD8 exhausted T cells | Lung cancer | FABP5 | 2.12 | logFC |
| 2994204 | CD8 exhausted T cells | Lung cancer | UBE2L6 | 2.11 | logFC |
| 2994204 | CD8 exhausted T cells | Lung cancer | MYO7A | 2.11 | logFC |
| 2994204 | CD8 exhausted T cells | Lung cancer | PRDX3 | 2.1 | logFC |
| 2994204 | CD8 exhausted T cells | Lung cancer | DDIT4 | 2.1 | logFC |
| 2994204 | CD8 exhausted T cells | Lung cancer | STMN1 | 2.09 | logFC |
| 2994204 | CD8 exhausted T cells | Lung cancer | CDK2AP2 | 2.09 | logFC |
| 2994204 | CD8 exhausted T cells | Lung cancer | VCAM1 | 2.08 | logFC |
| 2994204 | CD8 exhausted T cells | Lung cancer | SNAP47 | 2.07 | logFC |
| 2994204 | CD8 exhausted T cells | Lung cancer | PSMB3 | 2.06 | logFC |
| 2994204 | CD8 exhausted T cells | Lung cancer | ISG15 | 2.06 | logFC |
| 2994204 | CD8 exhausted T cells | Lung cancer | HLA-DRB5 | 2.05 | logFC |
| 2994204 | CD8 exhausted T cells | Lung cancer | CKS2 | 2.04 | logFC |
| 2994204 | CD8 exhausted T cells | Lung cancer | TNIP3 | 2.04 | logFC |
| 2994204 | CD8 exhausted T cells | Lung cancer | CD7 | 2.04 | logFC |
| 2994204 | CD8 exhausted T cells | Lung cancer | PSMD4 | 2.03 | logFC |
| 2994204 | CD8 exhausted T cells | Lung cancer | ATP6V1C2 | 2.03 | logFC |
| 2994204 | CD8 exhausted T cells | Lung cancer | PSMD8 | 2.01 | logFC |
| 2994204 | CD8 exhausted T cells | Lung cancer | HLA-DRB6 | 2.01 | logFC |

**Supplementary figures**


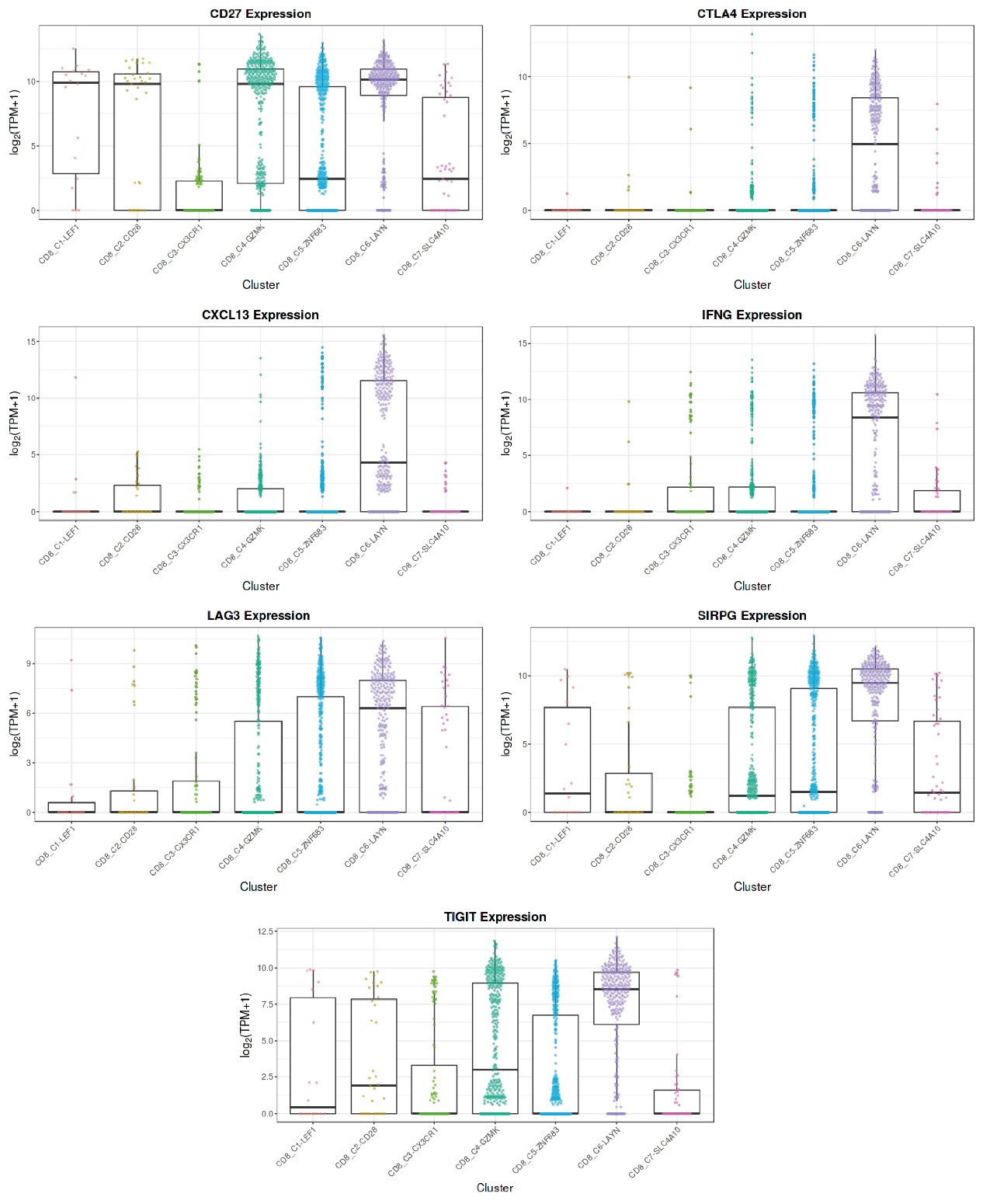


**Supplementary figure 1. The expression levels of seven genes of royalblue module in CD8 T cells.** CD27, CTLA4, CXCL13, IFNG, LAG3, SIRPG and TIGIT were highly expressed in CD8_C6-LAYN (exhausted CD8+ T cells).


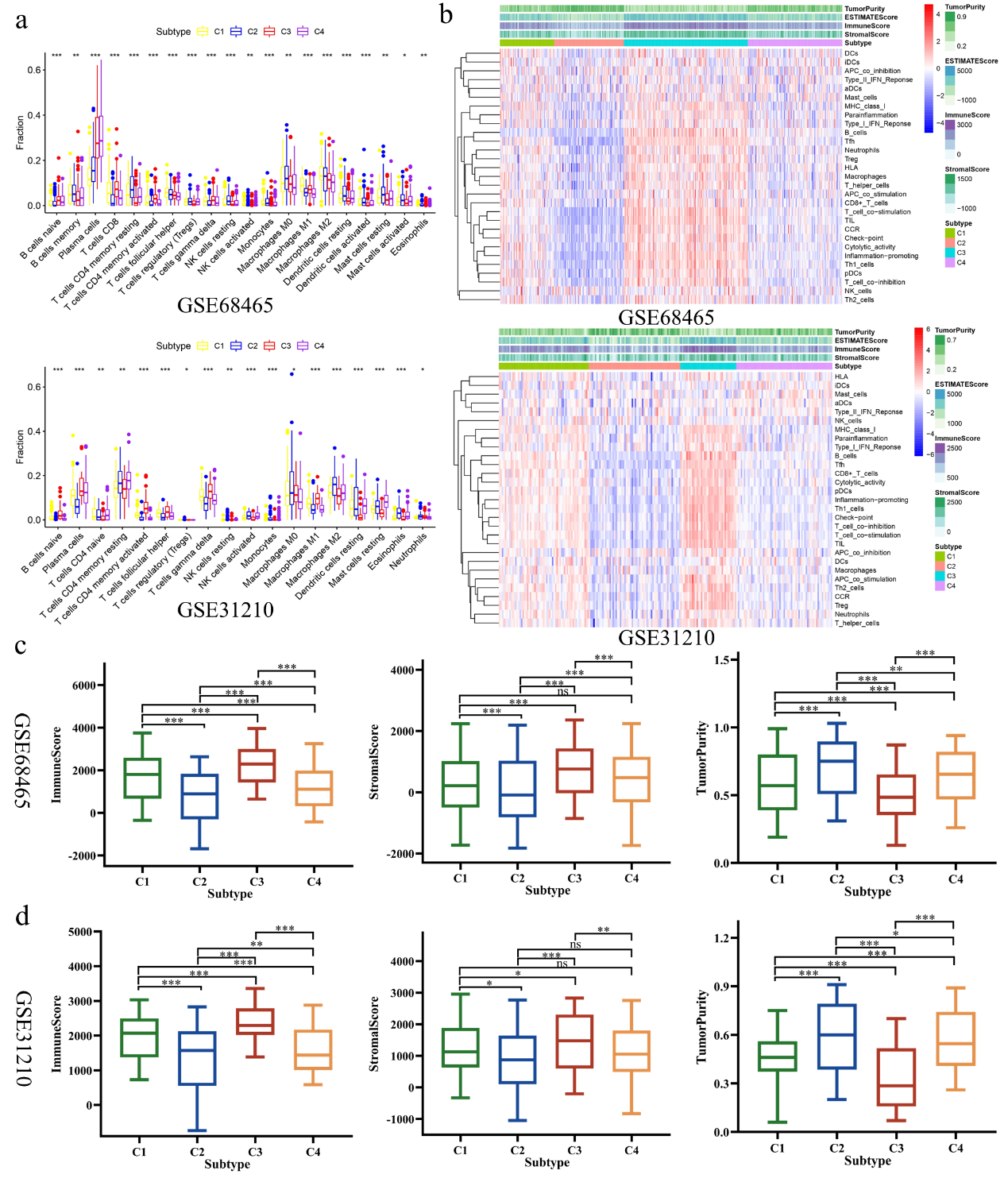


**Supplementary figure 2. Immune components of four immune subtypes** **in GSE68465 and GSE31210 cohorts.** (A) Heatmap of 29 immune gene sets for four immune subtypes in GSE68465 and GSE31210 cohorts. (B) Immune score of four immune subtypes in GSE68465 and GSE31210 cohorts. (C) Stromal score of four immune subtypes in GSE68465 and GSE31210 cohorts. (D) Tumor purity of four immune subtypes in GSE68465 and GSE31210 cohorts.


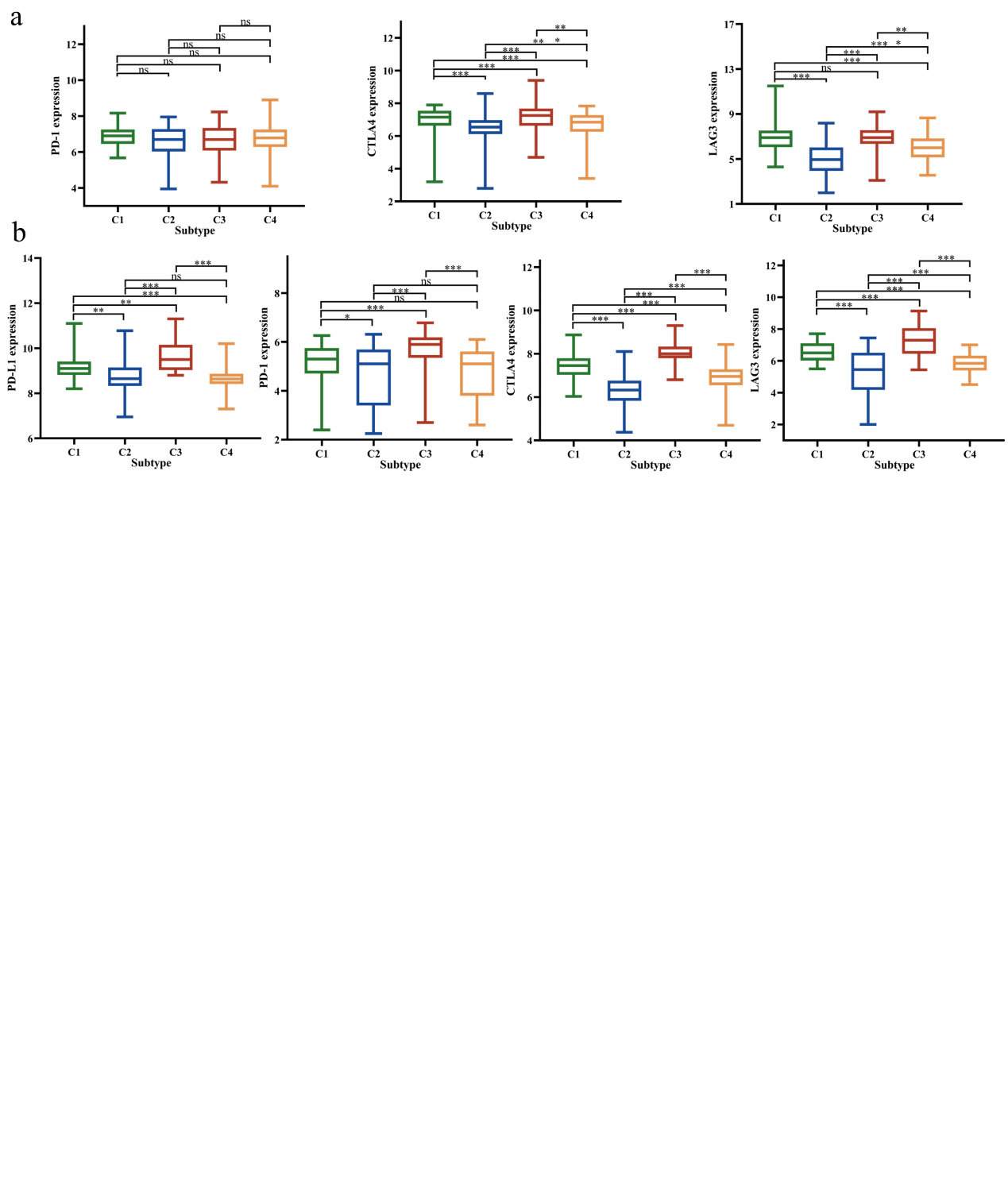


**Supplementary figure 3.** (a) Expression levels of PD-1, CTLA4 and LAG3 in four immune subtypes of GSE68465 cohort. (b) Expression levels of PD-1, PD-L1, CTLA4 and LAG3 in four immune subtypes of GSE31210 cohort.


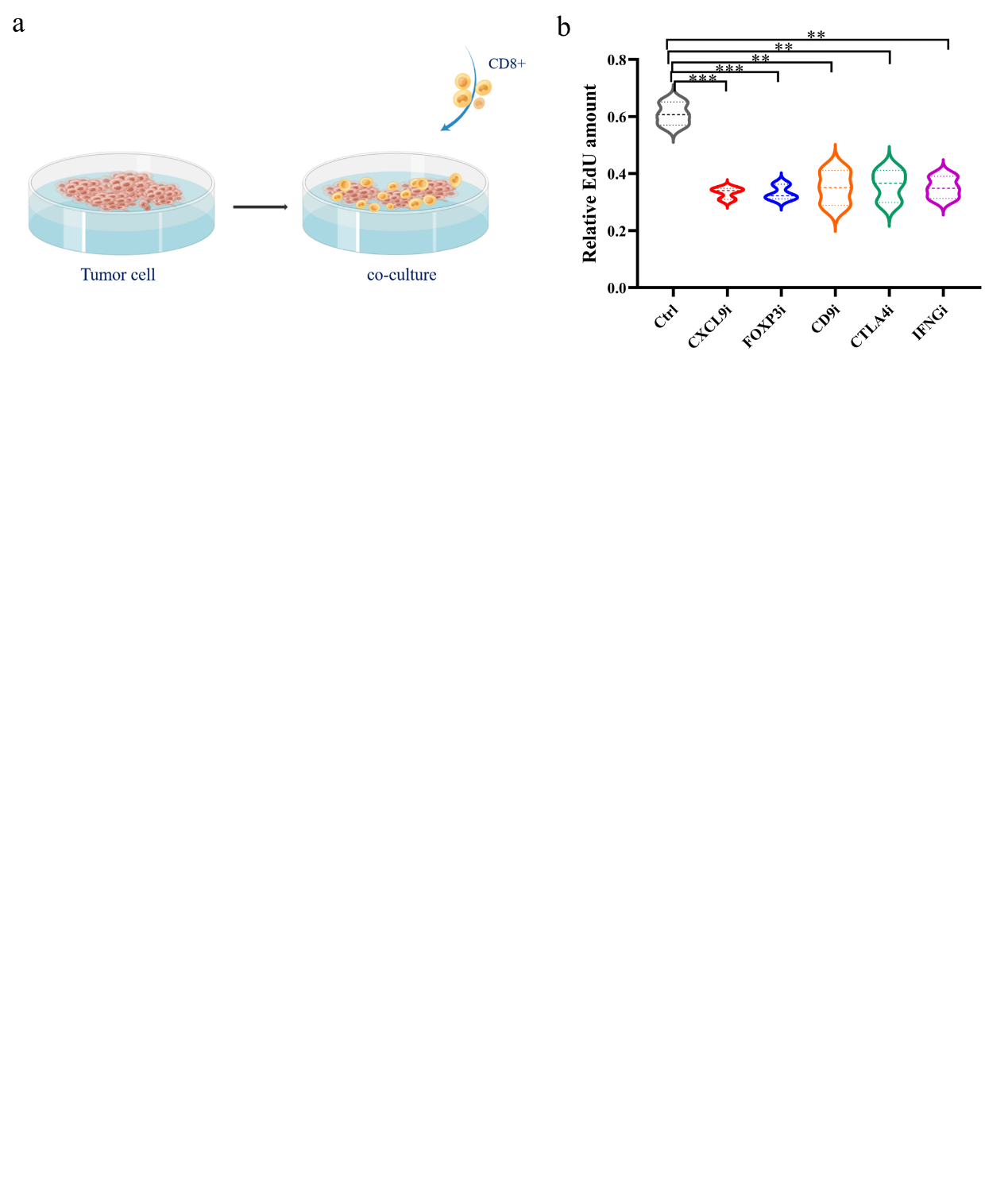


**Supplementary figure 4.** (a) Model of lung adenocarcinoma A549 cells co-culture with CD8+T cells. (b) Proliferation assays. 786-O cells were transfected with the indicated materials. Transfected cells were analyzed by EdU proliferation assays (n=3). A549 cells were co-cultured with CD8+T cells and exposed to inhibitors of these five hub genes for 24 hours, respectively. After that, the treated A549 cells were analyzed by EdU assay.
